# Sustained pain improvement and IL-12-related biomarker changes in rheumatoid arthritis patients initiating baricitinib or TNF inhibitor therapy

**DOI:** 10.1016/j.ero.2026.100259

**Published:** 2026-10-19

**Authors:** Christine Behrendt, Khetam Ghannam, Monika Brunner-Weinzierl, Anke Lux, André Wille, Mandy Gläß, Eugen Feist

**Affiliations:** 1 Experimental Rheumatology, Otto-von-Guericke University Magdeburg, Magdeburg, Germany; 2 Experimental Pediatrics, Otto-von-Guericke University Magdeburg, Magdeburg, Germany; 3 Institute of Medical Data Science, Otto-von-Guericke University Magdeburg, Magdeburg, Germany; 4 Rheumatology and Clinical Immunology, Helios Clinic Vogelsang-Gommern, Gommern, Germany

## Abstract

**Objectives:**

The Janus kinase (JAK) inhibitor baricitinib has demonstrated analgesic effects that may extend beyond anti-inflammatory mechanisms. This study investigated longitudinal pain trajectories and their association with interleukin (IL)-6 and IL-12 changes in patients with rheumatoid arthritis (RA) initiating baricitinib or tumour necrosis factor inhibitor (TNFi) therapy.

**Methods:**

In this single-centre prospective study, 60 patients with RA initiating baricitinib (n = 29) or TNFi therapy (n = 31) were followed at baseline, 2 weeks and 3 months. Healthy donors (n = 15) served as a biomarker reference. IL-6, IL-12p40, and IL-12p70 were measured using Enzyme-Linked Immunosorbent Assay (ELISA) and multiplex technology. Pain was assessed using numerical rating scales, and disease activity and function using 28-joint Disease Activity Score–erythrocyte sedimentation rate and Health Assessment Questionnaire Disability Index. Analyses were within-group and exploratory; adjusted mixed-effects models assessed between-group trajectories.

**Results:**

Within both groups, disease activity, function, and all 3 pain domains improved significantly over 3 months. Under baricitinib, average pain and swollen joint count (SJC) improved further between 2 weeks and 3 months, whereas TNFi showed earlier improvement at 2 weeks. IL-6, IL-12p40, and IL-12p70 showed within-group reductions under baricitinib, reaching significance at 3 months. Adjusted models showed no robust between-group differences.

**Conclusions:**

In this small prospective pilot cohort, both therapies were associated with early, clinically relevant pain improvement, with continued improvement in average pain and SJC under baricitinib. This sustained response was accompanied by within-group reductions in IL-6 and, notably, the IL-12 subunits, a JAK/tyrosine kinase(TYK)-linked pathway that has been less explored in relation to RA pain. These exploratory observations are hypothesis-generating and warrant confirmation in larger, adequately powered studies.

**WHAT IS ALREADY KNOWN ON THIS TOPIC**

• Pain may improve rapidly after initiation of targeted therapy in rheumatoid arthritis (RA), although symptom trajectories are not fully explained by inflammatory measures alone. In the pathogenesis of RA, neuroimmune signalling plays a role potentially causing chronic pain and difficult-to-treat disease.
• Compared with adalimumab, Janus kinase (JAK) inhibition by baricitinib has been associated with greater pain improvement in the RA-BEAM trial, a randomized, double-blind, placebo- and adalimumab-controlled phase 3 trial in patients with rheumatoid arthritis and an inadequate response to methotrexate [1,2]; however, the underlying mechanisms remain incompletely understood.

**WHAT THIS STUDY ADDS**

• Within both groups, pain improved early. Under baricitinib, average pain and swollen joint count showed further within-group improvement between 2 weeks and 3 months, indicating a prolonged analgesic and anti-inflammatory effect.
• This sustained response was accompanied by within-group reductions in interleukin (IL)-12p40 and IL-12p70, a pathway partly linked to JAK/tyrosine kinase (TYK) signalling. By tracking these 2 interleukins separately and longitudinally, rarely done in real-world RA, we observed within-group reductions under baricitinib and distinct behaviour between the 2 subunits, supporting IL-12 as an exploratory biomarker warranting further study.

**HOW THIS STUDY MIGHT AFFECT RESEARCH, PRACTICE OR POLICY**

• Early pain improvement in RA may be only partly reflected by conventional disease activity measures.
• IL-6 and IL-12-related pathways may warrant further evaluation as exploratory biomarkers during targeted therapy in RA, ideally assessing IL-12p40 and IL-12p70 separately rather than as a composite signal.
• Larger, adequately powered studies are needed to confirm these within-group signals and to clarify whether JAK inhibition and tumour necrosis factor blockade differ in their cytokine trajectories in RA.

Alt-text: Unlabelled box dummy alt text

## INTRODUCTION

Pain is one of the most burdensome symptoms of rheumatoid arthritis (RA) and remains a major determinant of impaired quality of life, physical function and treatment satisfaction [3,4]. Although pain in RA is closely linked to inflammation, pain intensity does not always parallel changes in composite disease activity measures [5,6]. In addition to inflammatory nociceptive pain, noninflammatory mechanisms such as peripheral and central sensitisation may contribute to persistent pain in RA [[5], [6], [7], [8], [9]].

Targeted therapies may therefore differ not only in their anti-inflammatory efficacy but also in the temporal profile of symptom improvement [1,10]. Tumour necrosis factor inhibitors (TNFi) are established treatment options in RA, whereas Janus kinase inhibitors (JAKi) such as baricitinib modulate intracellular signalling downstream of multiple cytokine receptors [[11], [12], [13]]. TNFi were selected as the comparator because they represent the established standard-of-care biologic class in RA [14,15] and act through an extracellular mechanism [16] distinct from intracellular JAK inhibition [17], providing a clinically relevant real-world contrast. Previous clinical and experimental work has suggested that baricitinib may provide rapid symptomatic benefit and may reduce pain to an extent not fully explained by inhibition of inflammatory pathways as reflected by conventional inflammatory markers alone [2,18].

Among candidate biomarkers relevant to inflammatory and pain-related pathways, interleukin (IL)-6 is of particular interest, as it represents a major JAK-dependent inflammatory pathway in RA and a plausible mediator of the early clinical effects of JAKi [17,[19], [20], [21]]. The IL-12 pathway may also be relevant in this context. IL-12 signals through JAK2/TYK2-signal transducer and activator of transcription 4(STAT4) pathways and has been linked to inflammatory activity in RA [[22], [23], [24]]. Although therapeutic targeting of the IL-12/23 axis has not shown significant clinical efficacy in RA [25], IL-12 may still serve as an informative biomarker of immune pathway modulation, particularly when assessed as IL-12p40 and IL-12p70, which differ in biological function and regulation [26]. IL-12 was therefore included as an exploratory marker that may reflect JAK-related immune modulation distinct from expected IL-6-related effects.

The aim of this study was to characterise longitudinal, within-group pain trajectories in patients with RA initiating baricitinib or TNFi therapy and to examine concurrent within-group changes in IL-6, IL-12p40, and IL-12p70 as exploratory biomarkers of JAK-related immune modulation.

## METHODS

### Study design and setting

This was a single-centre, investigator-initiated, prospective real-world observational study conducted at the Rheumatology Specialist Clinic Vogelsang/Gommern, Germany. Clinical assessments and biospecimen sampling were performed on site, and laboratory analyses were performed at the study site and University Medicine Magdeburg. The study was approved by the Ethics Committee of Otto-von-Guericke University Magdeburg (101/21) and conducted in accordance with the Declaration of Helsinki and Good Clinical Practice. All participants provided written informed consent before inclusion. Enrolment took place from December 8, 2021 to August 24, 2023.

### Patient population

A total of 60 patients (Fig. 1) with RA starting targeted therapy in routine care were included: 29 received baricitinib and 31 TNFi (Table 1 [Figs 2-4]). Eligible patients were aged ≥18 years and fulfilled the 2010 American College of Rheumatology (ACR)/European Alliance of Associations for Rheumatology (EULAR) classification criteria for RA [27]. In the TNFi group, etanercept was used in 84% and adalimumab in 16%. The 2 treatment groups were broadly comparable in terms of age, sex distribution, baseline disease activity, and functional impairment but differed notably in prior biologic exposure (Table 1). In addition, 15 healthy donors without known inflammatory rheumatic disease provided a single blood sample. These donors served solely as a descriptive biomarker reference and were not matched to the patient cohort or included in any longitudinal or between-group statistical analyses. The majority of patients were seropositive (Table 1). As this was a real-world study, treatment allocation was based on clinical decision- making and prior treatment history rather than randomisation. No formal exclusion criteria beyond standard contraindications to the prescribed therapies and inability to provide informed consent were applied. Blood samples were obtained at baseline, after 2 weeks, and after 3 months. Analyses were based on available data; missing values were not imputed. Concomitant medication is shown in Table 1.

**Figure 1:**
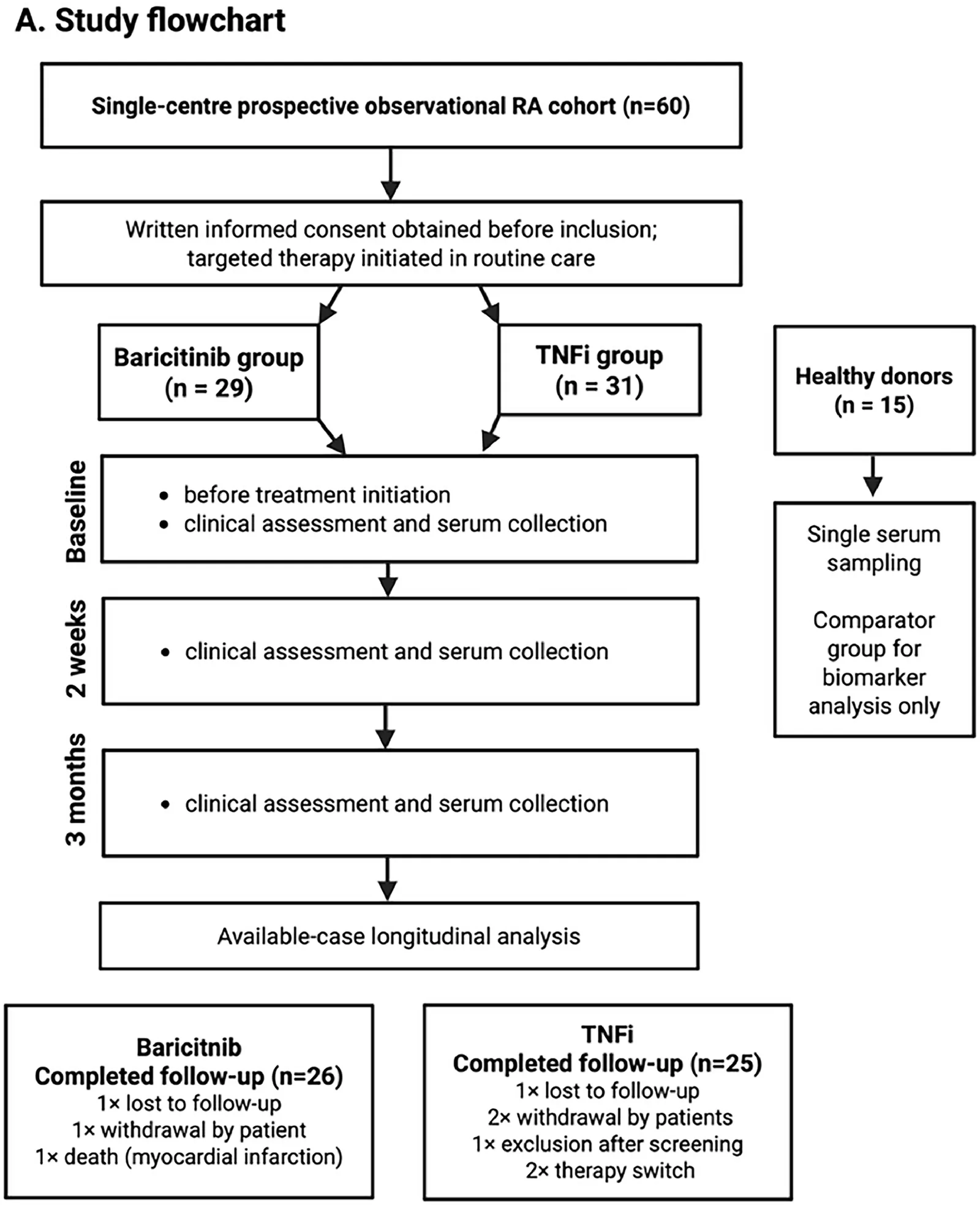
Study flowchart of a single-centre, prospective observational cohort of patients with rheumatoid arthritis (RA). After written informed consent, patients initiated baricitinib or tumour necrosis factor inhibitor (TNFi) therapy in routine care and were followed at baseline, 2 weeks and 3 months with clinical assessment and serum collection. Healthy donors were included as a comparator group for biomarker analyses and provided a single serum sample. Analyses were performed using available cases. Numbers of patients completing follow-up and reasons for noncompletion are shown.

**Table 1:** Baseline characteristics of the study population

Characteristic	All patients (n = 60)	Baricitinib (n = 29)	TNF inhibitor (n = 31)	Healthy donors (n = 15)
Female, %	73.33	72.41	74.19	73.33
Male, %	26.67	27.59	25.81	26.67
Age, mean ± SD, y	59 ± 11.3	58 ± 10.9	61 ± 11.7	44.0 ± 9.37
Current smokers, %	37.50	31.0	44.4	40.00
History of allergy, %	35.59	27.59	41.94	53.3
Disease duration, mean ± SD, y	8.34 ± 8.5	10.26 ± 9.95	6.49 ± 6.48	—
ESR, mean ± SD, mm/h	30 ± 21.1	27.8 ± 21.8	32.2 ± 20.8	—
IL-6, median (IQR), pg/mL	71.52 (24.79-100.73)	89.94 (53.82-115.74)	44.64 (13.36-88.84)	—
IL-12p40, median (IQR), pg/mL	804.71 (224.32-2978.00)	1122.92 (407.18-4438.47)	352.73 (203.62-1738.89)	224.32 (113.68-385.63)
IL-12p70, median (IQR), pg/mL	4.92 (1.97-12.97)	7.16 (3.47-22.18)	2.35 (1.69-6.73)	1.50 (1.17-2.35)
Anti-CCP and/or RF positive, %	76.67	93.10	61.9	—
Tender joint count, mean ± SD	10.6 ± 7.4	9.4 ± 6.5	11.6 ± 8.1	—
Swollen joint count, mean ± SD	4.9 ± 4.7	4.2 ± 3.2	5.6 ± 5.8	—
DAS28-ESR, mean ± SD	5.3 ± 1.3	5.0 ± 1.4	5.6 ± 1.1	—
HAQ-DI, mean ± SD	1.16 ± 0.69	1.13 ± 0.75	1.19 ± 0.64	—
Current pain NRS, mean ± SD	5.6 ± 2.2	5.4 ± 2.2	5.9 ± 2.3	—
Average pain NRS, mean ± SD	6.2 ± 1.9	6.0 ± 2.0	6.4 ± 1.7	—
Worst pain NRS, mean ± SD	7.5 ± 2.0	7.1 ± 2.2	8.0 ± 1.7	—
Patient global assessment, mean ± SD	6.4 ± 1.8	5.9 ± 1.9	6.8 ± 1.7	—
Current glucocorticoid use, %	63.3	69.0	58.1	—
Current methotrexate use, %	26.7	24.1	29.0	—
Current opioid use, %	5.0	10.3	0	—
Current NSAID use, %	48.3	62.1	35.5	—
Previous biologic therapy, %	43.3	75.9	12.9	—
Somatoform pain disorder, %	8.6	3.6	13.3	—
DASS depression, mean ± SD	6.8 ± 4.8	7.7 ± 5.3	5.9 ± 4.1	—
DASS anxiety, mean ± SD	4.0 ± 4.0	4.2 ± 4.4	3.8 ± 3.5	—
DASS stress, mean ± SD	7.3 ± 5.0	8.3 ± 5.7	6.3 ± 4.1	—
Central analgesic use (nonopioid), %	3.3	0	6.5	—

Anti-CCP, anticyclic citrullinated peptide antibodies; DAS28-ESR, Disease Activity Score in 28 joints using erythrocyte sedimentation rate; DASS, Depression Anxiety Stress Scales; ESR, erythrocyte sedimentation rate; HAQ-DI, Health Assessment Questionnaire Disability Index; IL, interleukin; NRS, numerical rating scale; NSAID, nonsteroidal anti-inflammatory drug; RF, rheumatoid factor; TNFi, tumour necrosis factor inhibitor.

Values are presented as mean ± SD, median (IQR) or percentage, unless otherwise indicated. Biomarker values are shown for available baseline serum samples only; sample numbers therefore differ from the full clinical cohort. Healthy donors served as a comparator group for biomarker analyses and were not part of the clinical cohort. An em dash indicates not applicable or not assessed. Baseline values shown here refer to the full baseline cohort; slightly smaller sample sizes were used for longitudinal within-group comparisons (Figs 2-4), as these were restricted to patients with available paired follow-up data.

### Patient and public involvement

Patients and/or the public were not involved in the design, conduct, reporting, or dissemination plans of this research.

### Clinical assessments

Pain intensity was assessed at all 3 time points using the German Pain Questionnaire of the German Pain Society [28]. From this instrument, numeric rating scale (NRS, 0-10) scores for current, average, and worst pain [29,30] were assessed longitudinally. Baseline depression, anxiety, and stress scores (DASS, rated on a 4-point Likert scale: 0 = ‘did not apply to me at all’ to 3 = ‘applied to me very much or most of the time’) were used to characterise the cohort (Table 1) and to contextualise the observed pain trajectories [31,32]. In exploratory analyses, a reduction of ≥2 NRS points was considered clinically relevant pain improvement [33]. Functional impairment was assessed using the Health Assessment Questionnaire Disability Index (HAQ-DI), with higher scores indicating greater disability. A reduction of >0.22 points was considered clinically relevant functional improvement [34]. For descriptive analyses, 28-joint Disease Activity Score–erythrocyte sedimentation rate (DAS28-ESR) values were categorised using established thresholds, with values <3.2 indicating low disease activity and values <2.6 representing the lowest disease activity category [35,36]. Adverse events (AEs) were recorded throughout the observation period at routine follow-up visits and classified descriptively as AEs or serious adverse events (SAEs). Owing to the observational study design, safety analyses were descriptive. Comorbid somatoform pain disorders, the diagnostic category that includes fibromyalgia, were recorded from patients’ existing diagnoses at baseline.

### Biomarker analyses

Serum concentrations of IL-12p40 and IL-12p70 were measured longitudinally in patients and once in healthy donors, whereas IL-6 was measured in patient samples only. IL-12p40 and IL-12p70 were determined using Human IL-12 p40 DuoSet ELISA (R&D Systems, catalogue no. DY1240-05) and Human IL-12 p70 DuoSet ELISA (R&D Systems, catalogue no. DY1270-05), respectively, according to the manufacturer’s instructions. IL-6 was measured using the Bio-Plex Pro Human Chemokine IL-6 Set (Bio-Rad; catalogue no. 171BK29MR2) based on Bio-Plex multiplex technology.

### Outcomes

The primary objective was to describe the longitudinal course of pain under baricitinib and TNFi therapy. Secondary clinical outcomes were changes in DAS28-ESR and HAQ-DI over time. Biomarker outcomes included longitudinal changes in serum IL-12p40, IL-12p70, and IL-6 concentrations. Exploratory analyses examined longitudinal changes in pain, disease activity, and biomarkers within each group. No formal between-group comparison was prespecified.

### Statistical analysis

As data were not normally distributed, nonparametric methods were used throughout. Within-group changes over time were analysed using the Wilcoxon signed-rank test for paired samples. Associations between continuous variables were assessed using Spearman’s rank correlation. Results are presented descriptively using medians, means, SDs, and proportions where appropriate. Because biomarker distributions were strongly right-skewed, the text emphasises distribution-aware interpretation rather than relying solely on mean values. Missing values were not imputed. Boxplots display all available observations at each time point, whereas longitudinal comparisons were based on paired observations available for the respective interval. All analyses were exploratory, and *P* values were interpreted descriptively. Within-group pairwise comparisons were corrected for multiple testing using the Holm-Bonferroni method. To address the nonrandomised allocation, we additionally fitted adjusted linear mixed-effects models for each outcome, with a random intercept per patient (accommodating the repeated measurements and using all available observations under a maximum-likelihood framework), fixed effects for group, time and their interaction, and adjustment for prior biologic exposure, glucocorticoid, and nonsteroidal anti-inflammatory drug (NSAID) use (cytokines log-transformed). Group-by-time interactions were tested by likelihood-ratio tests and interpreted descriptively as exploratory analyses; the final models were refitted by restricted maximum likelihood to obtain estimated marginal means with 95% CIs.

As the TNFi group comprised predominantly etanercept, a sensitivity analysis repeating the adjusted models after excluding adalimumab-treated patients was performed.

For the cytokines, the longitudinal models were additionally repeated with the log-transformed baseline concentration as a covariate, to assess the influence of baseline imbalance and regression to the mean. The adjusted models were additionally repeated with disease duration as a further covariate.

## RESULTS

### Baseline characteristics

The cohort was predominantly female (73%), with a mean age of 59 years, and presented with active disease and moderate-to-severe pain at baseline (Table 1). Most patients were seropositive (77% anticyclic citrullinated peptide antibodies and/or rheumatoid factor positive). The treatment groups were broadly comparable in age, sex, and baseline disease activity but differed notably in prior treatment: patients in the baricitinib group more frequently had prior biologic exposure (75.9% vs 12.9%) and more often used glucocorticoids (69.0% vs 58.1%) and NSAIDs (62.1% vs 35.5%) at baseline than those in the TNFi group. Biomarker data from 15 healthy donors were available for comparison only (Table 1). Glucocorticoid use declined over follow-up in both groups, and among patients remaining on glucocorticoids the median dose did not increase (5 mg/d prednisolone equivalent at 3 months; Supplementary Table S1). More patients reduced than increased their glucocorticoid dose (baricitinib: 15 vs 3; TNFi: 13 vs 3), and no systematic dose escalation occurred.

### Clinical outcomes

#### Pain

Mean current pain changed from 5.4 to 4.3 (n = 24; *P =* .020) and to 3.4 (n = 22; *P =* .020) under baricitinib and from 5.9 to 4.5 (n = 27; *P =* .011) and to 4.2 (n = 25; *P =* .001) under TNFi therapy. No additional significant changes were observed between 2 weeks and 3 months in either group (baricitinib *P =* .341; TNFi *P =* .207) (Fig 2). Under baricitinib, mean average pain declined from 6.0 to 5.3 (n = 24; *P =* .041) and to 3.8 (n = 22; *P =* .004), whereas under TNFi therapy it changed from 6.4 to 4.9 (n = 27; *P =* .010) and to 4.6 (n = 25; *P =* .002). An additional significant reduction between 2 weeks and 3 months was observed only in the baricitinib group (n = 23; *P =* .045) (Fig 2). Mean worst pain changed from 7.1 to 6.9 (n = 23; *P =* .121, not significant) and to 4.7 (n = 21; *P =* .002) under baricitinib and from 8.0 to 6.1 (n = 27; *P =* .014) and to 5.3 (n = 25; *P <* .001) under TNFi therapy. Between 2 weeks and 3 months, worst pain decreased significantly in both groups (baricitinib: n = 23, *P =* .025; TNFi: n = 26, *P =* .039) (Fig 2).

**Figure 2:**
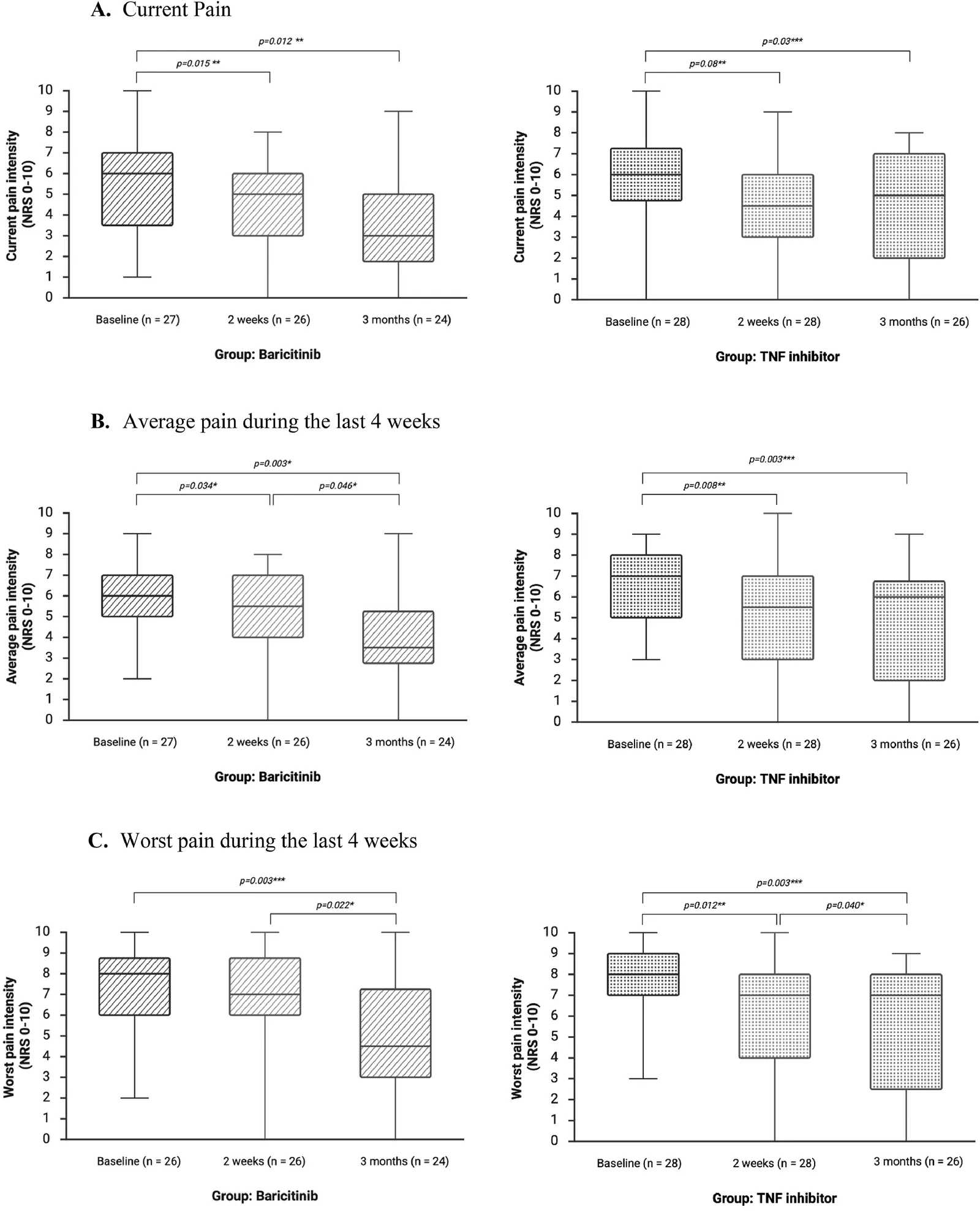
Longitudinal changes in current, average and worst pain under baricitinib and TNFi therapy. Current pain (A), average pain (B), and worst pain (C) at baseline, 2 weeks, and 3 months in patients initiating baricitinib or TNFi therapy. Pain was assessed using an 11-point NRS (0-10). Boxplots show median and IQR; whiskers represent minimum and maximum values. The number of observations per time point is indicated below each box. Within-group comparisons are based on paired Wilcoxon signed-rank tests: * *P <* .05, ** *P <* .01, and *** *P <* .001; exact *P* values are shown for comparisons with *P <*.10 that did not reach statistical significance after correction. Multiple corrections were done using the Holm-Bonferroni method. Missing values were not imputed. NSR, numerical rating scale; TNFi, tumour necrosis factor inhibitor.

#### Clinically relevant pain improvement

The proportion of patients achieving a clinically relevant improvement (≥2-point NRS reduction) from baseline is shown in Table 2. By 3 months, clinically relevant improvement in current, average, and worst pain was reached by 45.5%, 59.1%, and 61.9% of baricitinib-treated patients and 56.0%, 48.0%, and 64.0% of TNFi-treated patients, respectively. Proportions were comparable between groups at both follow-up visits (Fisher’s exact test, all *P* > .5). A further ≥2-point improvement between 2 weeks and 3 months occurred in 30% to 35% of baricitinib-treated and 31% to 42% of TNFi-treated patients across domains.

**Table 2:** Proportion of patients achieving clinically relevant pain improvement (≥2-point NRS reduction) from baseline

Pain domain	Time point	Baricitinib, n/N (%)	TNFi, n/N (%)	Fisher *P*
Current pain	2 wk	10/24 (41.7)	12/27 (44.4)	1.00
	3 mo	10/22 (45.5)	14/25 (56.0)	.564
Average pain	2 wk	9/24 (37.5)	13/27 (48.1)	.573
	3 mo	13/22 (59.1)	12/25 (48.0)	.561
Worst pain	2 wk	8/23 (34.8)	10/27 (37.0)	1.00
	3 mo	13/21 (61.9)	16/25 (64.0)	1.00

TNFi, tumour necrosis factor inhibitor.

Clinically relevant improvement defined as a ≥2-point reduction in the numerical rating scale (NRS) vs baseline. Denominators reflect available paired observations. Between-group comparisons by Fisher’s exact test; no comparison was prespecified.

#### DAS28-ESR

Disease activity decreased significantly in both treatment groups from baseline to 2 weeks and from baseline to 3 months (Fig 4). In the baricitinib group, mean DAS28-ESR declined from 5.0 at baseline to 4.2 at 2 weeks (n = 26; *P =* .022) and 3.3 at 3 months (n = 24; *P =* .003). In the TNFi group, corresponding values were 5.6, 4.2, and 4.1 (n = 25, *P =* .003 at 2 weeks; n = 26, *P =* .002 at 3 months). An additional significant reduction between 2 weeks and 3 months was observed in the baricitinib group but not the TNFi group (n = 24; *P =* .034) (Fig 4).

#### Objective inflammatory measures

Swollen joint count (SJC), an objective inflammatory measure, decreased significantly from baseline to 3 months in both groups (baricitinib 4.2-1.4; TNFi 5.6-2.0; both corrected *P* ≤ .001), with a further significant reduction between 2 weeks and 3 months only under baricitinib (*P =* .033). Tender joint count showed a comparable pattern, with continued reduction between 2 weeks and 3 months only under baricitinib (*P =* .013) (Supplementary Table S2). C-reactive protein (CRP)–based indices were not used as an additional activity measure; as JAK inhibition can lower CRP through partial inhibition of IL-6 signalling, which may not fully parallel clinical joint activity [37,38]. Swollen and tender joint counts were prioritised as a treatment-independent inflammatory readout.

#### HAQ-DI

Functional impairment (HAQ-DI) improved significantly only in the TNFi group. Under TNFi, a significant reduction was observed at 2 weeks (n = 26; *P =* .015) and was sustained at 3 months (n = 26; *P =* .015). Under baricitinib, no significant change was detected at any time point after correction (2 weeks: n = 24, *P =* .127; 3 months: n = 23, *P =* .127). No significant further change occurred between 2 weeks and 3 months in either group (baricitinib: n = 24, *P =* .483; TNFi: n = 24, *P =* .553). Mean HAQ-DI scores were 1.13 at baseline, 1.12 at 2 weeks, and 1.06 at 3 months under baricitinib, and 1.19, 0.85, and 0.97 under TNFi, respectively.

### Biomarker outcomes

#### IL-6

IL-6 concentrations were elevated at baseline in both treatment groups. In the baricitinib group, median IL-6 showed a nonsignificant decrease from 89.9 pg/mL at baseline to 66.2 pg/mL at 2 weeks (n = 25; *P =* .062) and decreased significantly to 56.5 pg/mL at 3 months (n = 24; *P =* .015), after correction for multiple comparisons. In the TNFi group, median IL-6 changed from 44.6 to 25.9 at 2 weeks (n = 27; *P =* .156) and to 30.0 pg/mL at 3 months (n = 26; *P =* .118), indicating nonsignificant downwards trends (Fig 3).

**Figure 3:**
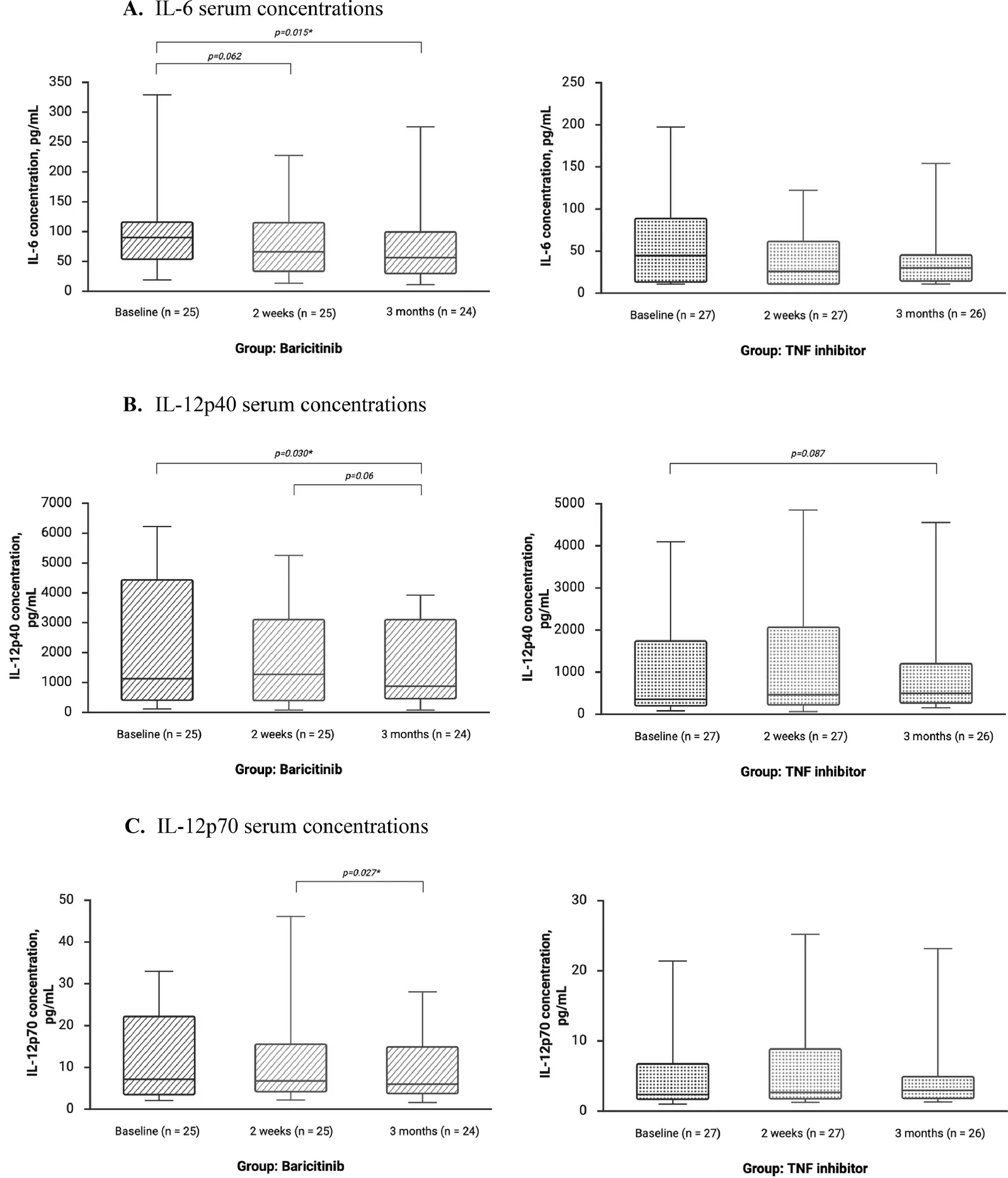
Longitudinal changes in IL-6, IL-12p40, and IL-12p70 under baricitinib and TNFi therapy. Serum IL-6 (A), IL-12p40 (B), and IL-12p70 (C) concentrations at baseline, 2 weeks, and 3 months in patients initiating baricitinib or TNFi therapy. Boxplots show median and IQR; whiskers represent the 5th and 95th percentiles. The number of observations per time point is indicated below each box. Within-group comparisons were performed using paired Wilcoxon signed-rank tests, and P values were adjusted for multiple comparisons using the Holm-Bonferroni method. Exact adjusted P values are shown for all comparisons with P < .10; * P < .05. Missing values were not imputed. IL, interleukin; TNFi, tumour necrosis factor inhibitor.

**Figure 4:**
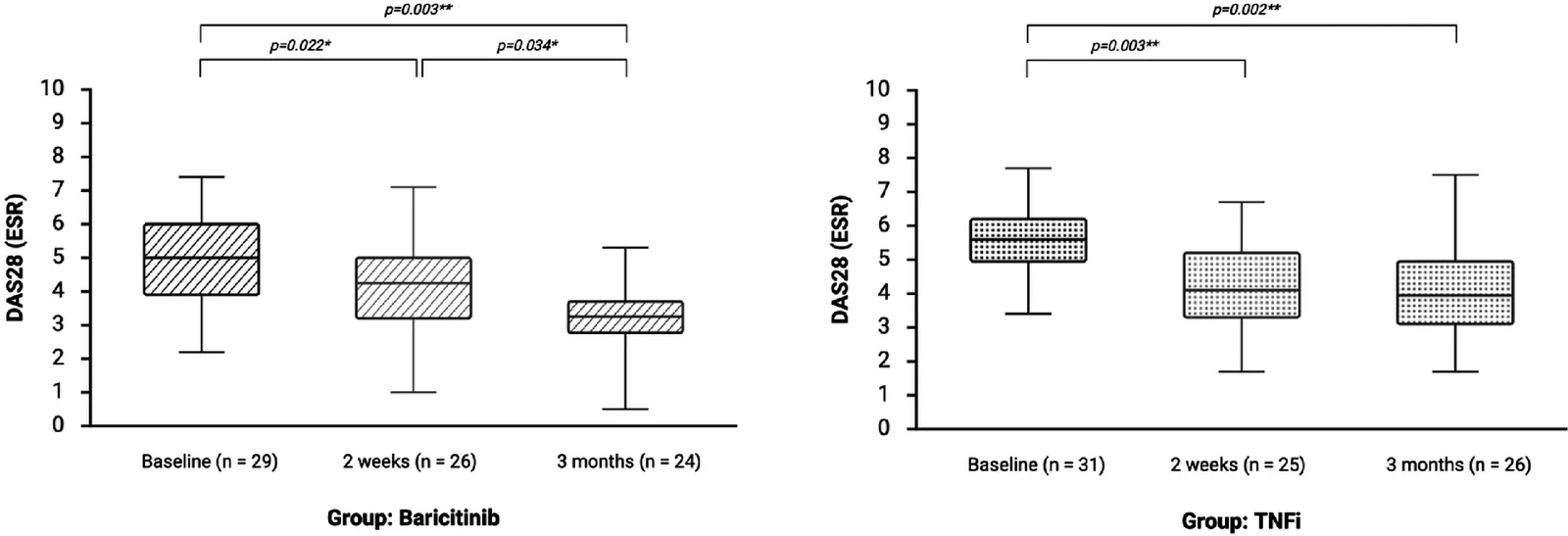
Longitudinal changes in DAS28-ESR under baricitinib and TNFi therapy. DAS28-ESR at baseline, 2 weeks, and 3 months in patients with rheumatoid arthritis initiating baricitinib or TNFi therapy. Boxplots show median and IQR; whiskers represent minimum and maximum values. The number of observations per time point is indicated below each box. Within-group comparisons were performed using paired Wilcoxon signed-rank tests, and P values were adjusted for multiple comparisons using the Holm-Bonferroni method. Exact adjusted P values are shown; * P < .05, ** P < .01. Missing values were not imputed. DAS28-ESR, Disease Activity Score in 28 joints using the erythrocyte sedimentation rate; TNFi, tumour necrosis factor inhibitor.

#### IL-12p40

IL-12p40 concentrations were lower in healthy donors than in patients with RA (Table 1). Baseline levels were higher in the baricitinib group than in the TNFi group (Table 1). In both treatment groups, IL-12p40 showed marked interindividual variability and right-skewed distributions. No significant changes were observed between baseline and 2 weeks in either group. From baseline to 3 months, IL-12p40 decreased significantly only in the baricitinib group (n = 24; *P =* .030); the reduction in the TNFi group did not reach significance after correction (n = 26; *P =* .087). No significant change was observed between 2 weeks and 3 months in either group after correction (baricitinib: n = 24, *P =* .060; TNFi: n = 26, *P =* .747) (Figure 3).

#### IL-12p70

IL-12p70 concentrations were lower in healthy donors than in patients with RA (Table 1). Among patients, baseline levels were higher in the baricitinib group than in the TNFi group (Table 1). No significant changes were observed between baseline and 2 weeks in either group. From baseline to 3 months, baricitinib showed a nonsignificant trend towards reduction, whereas TNFi did not. Between 2 weeks and 3 months, a significant decrease in IL-12p70 was observed only under baricitinib (n = 24, *P* = .027) (Fig 3).

Individual patient trajectories for IL-6, IL-12p40, and IL-12p70 are shown as spaghetti plots in Supplementary Figures S1 (baricitinib) and S2 (TNFi), illustrating the marked interindividual variability in cytokine responses.

### Exploratory biomarker-pain correlations

A total of 72 exploratory Spearman correlations were examined, relating IL-12p40 and IL-12p70 concentrations and their changes to the 3 pain domains across both treatment groups, individual visits, and change intervals. Only 1 reached nominal significance, and none remained significant after correction for multiple testing.

### Adjusted longitudinal analysis

All primary analyses were performed within groups; no between-group comparison was prespecified. To address the concern about confounding, we additionally fitted adjusted mixed-effects models including a group-by-time interaction. These exploratory models did not indicate robust between-group differences in trajectory; nominal interaction signals and adjusted estimated marginal means for both groups are provided in Supplementary Tables S3 and S4.

In a sensitivity analysis restricted to baricitinib- and etanercept-treated patients, the adjusted interaction results were essentially unchanged, and pain and DAS28-ESR trajectories for etanercept-treated patients differed from the pooled TNFi group by no >0.4 NRS points at any time point (Supplementary Table S5). After additionally adjusting the cytokine models for baseline concentration, the nominal between-group differences in cytokine trajectories were no longer apparent (interaction *P =* .13 for IL-6, *P =* .31 for IL-12p40, *P =* .09 for IL-12p70; Supplementary Table S6), consistent with a contribution of regression to the mean. Results were unchanged when disease duration was added as a further covariate.

### Safety

Overall, 15 patients (25.0%) experienced at least 1 AE during the study period, including 8 patients in the baricitinib group (27.6%) and 7 in the TNFi group (22.6%). A total of 22 AEs were recorded. The most common AEs were respiratory tract infections (including COVID-19 and influenza-like illness; n = 12) and skin or mucosal reactions (n = 8), whereas gastrointestinal complaints (n = 1) and urinary tract infections (n = 1) occurred only in the TNFi group. Three patients (5.0%) experienced an SAE. In the TNFi group, these included hospitalisation for evaluation of unintentional weight loss and surgery for suspected pleomorphic adenoma of the parotid gland. In the baricitinib group, one 67-year-old woman treated with baricitinib 4 mg experienced a fatal acute myocardial infarction in March 2022. Aside from arterial hypertension, she had no known cardiovascular comorbidities and was a nonsmoker; she had previously received biologic therapy, including the TNF inhibitor etanercept. Enrolment and the event occurred before the finalised European Medicines Agency (EMA) risk-minimisation recommendations and the corresponding Direct Healthcare Professional Communication (March 2023), which restrict JAK-inhibitor use in patients aged ≥65 years, current or former long-term smokers, and those with cardiovascular risk factors [39]. A causal relationship with baricitinib could not be established.

## DISCUSSION

In this prospective real-world study, both baricitinib and TNFi therapy were associated with early clinical improvement in RA, including reductions in disease activity and pain, consistent with the established efficacy of both treatment strategies [2,15]. However, the time-course of improvement varied between outcomes. Most notably, average pain continued to decline significantly between 2 weeks and 3 months in the baricitinib group, whereas this pattern was not observed in the TNFi group. These findings suggest that pain trajectories under targeted therapy may be heterogeneous and not fully captured by composite disease activity measures alone. This interpretation is broadly consistent with previous baricitinib trial data, including the RA-BEAM-trial, which identified pain as an important and responsive outcome under baricitinib treatment [1,18], although the present real-world data indicate continued pain reduction over time rather than formal evidence of faster pain relief. As the study was not designed for formal between-group inference, these observations are descriptive; they are nonetheless in line with the concept that patient-perceived symptom improvement and changes in composite disease activity do not necessarily occur in parallel [40,41]. The concurrent decline in SJC, an objective inflammatory measure independent of patient-reported pain, indicates that the observed improvements reflect genuine anti-inflammatory activity rather than analgesia alone, and mitigates the concern that DAS28-ESR is partly driven by pain. DAS28-ESR was chosen as the disease activity index because it incorporates directly examinable, objective joint parameters, including the swollen and tender joint counts, alongside a systemic marker, and was recorded consistently at all visits. Notably, the improvements in pain and disease activity occurred despite a reduction rather than an increase in glucocorticoid use, arguing against concomitant glucocorticoid changes as an explanation for the observed trajectories.

The biomarker analyses provide a complementary perspective. Baseline cytokine concentrations were numerically higher in the baricitinib group than in the TNFi group, particularly for IL-6 and IL-12p70. Because of this difference, regression to the mean may partly account for the larger apparent declines over time. Consistent with this, adjusting the longitudinal cytokine models for baseline concentration attenuated the between-group differences (Supplementary Table S6). In this nonrandomised setting, such differences may reflect variation in inflammatory burden, disease phenotype, or prior treatment exposure rather than treatment-specific effects alone [42,43].

IL-6 decreased significantly from baseline to 3 months in the baricitinib group; the reduction at 2 weeks did not reach significance after multiple comparison correction. Given the role of IL-6 in inflammatory signalling and nociceptive sensitisation [44], this pattern may be relevant to the sustained pain improvement observed with baricitinib. However, the observational design does not allow causal inference, and these findings should be interpreted as descriptive rather than mechanistic evidence. The most distinctive cytokine pattern was observed for the IL-12 subunits. IL-12p40 decreased significantly from baseline to 3 months only in the baricitinib group; the reduction in the TNFi group did not reach significance after multiple comparison correction. IL-12p70 showed an even more selective pattern, with a significant decrease between 2 weeks and 3 months only in the baricitinib group. The nonparallel behaviour of IL-12p40 and IL-12p70 is biologically plausible, as these analytes are functionally related but not interchangeable and are subject to different regulation, expression patterns, and biological stability [[45], [46], [47]].

The concurrent within-group decline in IL-12p40 and IL-12p70 under baricitinib is of interest because IL-12 signals in part through the JAK2/TYK2-STAT4 pathway [19,20], which was part of our rationale for examining these analytes. Existing literature links this axis to downstream proteolytic and neurotrophic mediators of nociception [48] such as furin and β-NGF [[49], [50], [51]], but as these were not measured here, we present a possible IL-12-pain link only as a hypothesis for dedicated mechanistic studies. Our biomarker panel focused a priori on IL-6 and the IL-12 subunits, selected for their relationship to JAK/STAT signalling; other cytokines with emerging roles in RA and pain, such as granulocyte-macrophage colony-stimulating factor (GM-CSF), were not measured and represent a relevant target for future studies.

### Limitations

Several methodological considerations should be noted. The nonrandomised design introduces possible confounding by indication: the baricitinib group had higher rates of prior biologic exposure and more frequent glucocorticoid and NSAID use, which may have influenced both symptom trajectories and biomarker profiles independently of treatment. To address this, we fitted adjusted mixed-effects models with a group-by-time interaction, which did not reveal robust between-group differences. The modest sample size and available-case analyses without imputation further limit the precision of estimates. The TNFi group comprised predominantly etanercept, and a formal subgroup analysis of the few adalimumab-treated patients (n = 5) was not feasible; however, a sensitivity analysis excluding these patients did not materially alter the findings, suggesting that pooling of TNFi agents did not drive the observed patterns. Biomarker distributions were highly variable and right-skewed, particularly for IL-12p40 and IL-6, constraining the interpretation of summary statistics. The healthy donors provided a single baseline sample as a descriptive reference only; they were younger than the patients and were not matched or included in the inferential analyses, so the patient-donor comparison should be interpreted with caution. The 3-month follow-up captures early and intermediate trajectories but not long-term persistence, which may diverge or converge between therapeutic classes over time. Despite these limitations, the study provides prospective real-world data across early and intermediate visits, combines patient-reported pain with disease activity and functional measures, and analyses IL-12p40 and IL-12p70 separately rather than as a single composite signal, revealing distinct patterns that a coarser dataset might have obscured. As a small pilot cohort, it was designed to generate hypotheses rather than to establish between-group effects; the findings accordingly require confirmation in larger, adequately powered, longer-term studies.

## CONCLUSION

In conclusion, both baricitinib and TNFi therapy were associated with early, clinically relevant improvement in RA, particularly in pain. In the baricitinib group, average pain and SJC continued to improve between 2 weeks and 3 months, accompanied by within-group reductions in the IL-12 subunits IL-12p40 and IL-12p70. These exploratory findings suggest that improvement in pain during targeted therapy may be only partly captured by conventional composite measures of disease activity. They also suggest that IL-12-related pathways may be worth investigating further. Larger, adequately powered and longer-term cohorts are needed to confirm these within-group signals and to determine whether they are sustained.

## CRediT authorship contribution statement

**Christine Behrendt:** Writing – review & editing, Writing – original draft, Visualization, Project administration, Investigation, Data curation. **Khetam Ghannam:** Validation, Methodology, Investigation, Formal analysis. **Monika Brunner-Weinzierl:** Investigation, Formal analysis, Data curation. **Anke Lux:** Validation, Formal analysis. **André Wille:** Supervision, Resources, Investigation, Data curation. **Mandy Gläß:** Supervision, Resources, Investigation, Data curation. **Eugen Feist:** Writing – review & editing, Validation, Supervision, Resources, Project administration, Methodology, Investigation, Funding acquisition, Data curation, Conceptualization.

## Competing interests

EF received honoraria as a speaker and adviser from AbbVie, AB2Bio, Alfasigma, AstraZeneca, BMS, Celgene, GSK, J&J, Lilly, Medac, Novartis, Pfizer, Roche/Chugai, Sanofi, Sobi, and UCB. All other authors declare no competing interests.

## Appendix Supplementary materials

Image, application 1

## Footnotes

Supplementary material associated with this article can be found in the online version at doi:10.1016/j.ero.2026.100259.
