# Supplementary material for: Sustained pain improvement and IL-12-related biomarker changes in rheumatoid arthritis patients initiating baricitinib or TNF inhibitor therapy
Source: EULAR Rheumatol Open. 2026 Oct 19;2(4):100259. doi: 10.1016/j.ero.2026.100259 (PMC13698054; doi:10.1016/j.ero.2026.100259)
Supplement: Supplementary file 1 [file mmc1.docx]

# SUPPLEMENTARY MATERIAL

**SUPPLEMENTARY TABLES**

**Supplementary Table S1. Glucocorticoid use and dose over follow-up**

| **Group** | **Visit** | **On glucocorticoid, n/N (%)** | **Median dose, users (mg/day)** | **Mean dose, all patients (mg/day)** |
| --- | --- | --- | --- | --- |
| **Baricitinib** | Baseline | 20/29 (69) | 5.0 | 6.8 |
|  | 2 weeks | 19/29 (66) | 5.0 | 4.1 |
|  | 3 months | 9/29 (31) | 5.0 | 2.8 |
| **TNFi** | Baseline | 18/31 (58) | 7.5 | 6.1 |
|  | 2 weeks | 16/31 (52) | 5.0 | 3.3 |
|  | 3 months | 11/31 (35) | 5.0 | 2.2 |
|  |  |  |  |  |
| **Dose change, baseline to 3 months** | | |  |  |
| **Group** | **Decreased** | **Increased** | **Unchanged** |  |
| **Baricitinib** | 15 | 3 | 11 |  |
| **TNFi** | 13 | 3 | 15 |  |

Concomitant glucocorticoid (prednisolone-equivalent) use and dose at each visit, by group. Median dose is shown for patients remaining on glucocorticoids.

**Supplementary Table S2. Swollen and tender joint count trajectories**

| **Measure** | **Group** | **Baseline** | **2 weeks** | **3 months** | **p (BL-3mo)** | **p (2wk-3mo)** |  |
| --- | --- | --- | --- | --- | --- | --- | --- |
| **SJC** | Baricitinib | 4.2 | 3.0 | 1.4 | 0.001 | 0.033 |  |
|  | TNFi | 5.6 | 2.2 | 2.0 | <0.001 | 0.517 |  |
| **TJC** | Baricitinib | 9.4 | 6.9 | 3.0 | <0.001 | 0.013 |  |
|  | TNFi | 11.6 | 5.8 | 5.2 | 0.006 | 0.383 |  |

Within-group changes in swollen joint count (SJC) and tender joint count (TJC). Means at baseline, 2 weeks and 3 months; p-values from paired Wilcoxon signed-rank tests, Holm-Bonferroni corrected within each group.

**Supplementary Table S3. Adjusted estimated marginal means (95% CI)**

| **Outcome** | **Time point** | **TNFi - mean** | **TNFi - 95% CI** | **Baricitinib - mean** | **Baricitinib - 95% CI** |
| --- | --- | --- | --- | --- | --- |
| **Current pain (NRS)** | Baseline | 6.0 | 5.0-7.0 | 5.5 | 4.4-6.5 |
|  | 2 weeks | 4.6 | 3.6-5.6 | 4.2 | 3.1-5.2 |
|  | 3 months | 4.1 | 3.0-5.1 | 3.4 | 2.3-4.5 |
| **Average pain (NRS)** | Baseline | 6.5 | 5.6-7.5 | 6.0 | 5.0-7.0 |
|  | 2 weeks | 5.0 | 4.1-6.0 | 5.0 | 4.0-6.1 |
|  | 3 months | 4.6 | 3.7-5.6 | 3.6 | 2.6-4.7 |
| **Worst pain (NRS)** | Baseline | 8.1 | 7.0-9.2 | 7.1 | 6.0-8.2 |
|  | 2 weeks | 6.2 | 5.1-7.3 | 6.6 | 5.4-7.7 |
|  | 3 months | 5.4 | 4.3-6.5 | 4.5 | 3.3-5.7 |
| **IL-6 (pg/mL)** | Baseline | 47.0 | 31.5-70.1 | 68.9 | 44.6-106.5 |
|  | 2 weeks | 32.3 | 21.6-48.2 | 52.3 | 33.8-80.8 |
|  | 3 months | 36.1 | 24.1-54.0 | 44.0 | 28.4-68.2 |
| **IL-12p40 (pg/mL)** | Baseline | 635 | 360-1120 | 857 | 461-1594 |
|  | 2 weeks | 727 | 412-1283 | 732 | 394-1360 |
|  | 3 months | 831 | 470-1468 | 685 | 368-1275 |
| **IL-12p70 (pg/mL)** | Baseline | 4.6 | 3.0-6.9 | 5.7 | 3.6-9.0 |
|  | 2 weeks | 5.1 | 3.3-7.7 | 5.9 | 3.7-9.4 |
|  | 3 months | 5.0 | 3.3-7.7 | 4.9 | 3.1-7.7 |

Model-based means from adjusted linear mixed-effects models (random intercept per patient; fixed effects for group, time and their interaction; adjusted for prior biologic exposure, glucocorticoid and NSAID use; refitted by REML). Covariates at sample means. Cytokines as geometric means (pg/mL); pain as NRS 0-10.

**Abbreviations**: EMM, estimated marginal means; CI, confidence interval; NRS, numerical rating scale.

**Supplementary Table S4. Adjusted group-by-time interaction models (exploratory)**

| **Outcome** | **Patients** | **Observations** | **Interaction p (LRT)** | **Covariate-block p (LRT)** |
| --- | --- | --- | --- | --- |
| **Current pain** | 58 | 159 | 0.924 | 0.722 |
| **Average pain** | 58 | 159 | 0.336 | 0.688 |
| **Worst pain** | 58 | 158 | 0.128 | 0.817 |
| **log IL-6** | 52 | 154 | 0.362 | 0.041 |
| **log IL-12p40** | 52 | 154 | 0.025 | 0.116 |
| **log IL-12p70** | 52 | 154 | 0.031 | 0.052 |
|  |  |  |  |  |

Adjusted linear mixed-effects models (random intercept per patient; adjusted for prior biologic exposure, glucocorticoid and NSAID use; cytokines log-transformed). The group-by-time interaction and the covariate block were each tested by likelihood-ratio test (maximum likelihood). No between-group comparison was prespecified; p-values are descriptive.

**Abbreviations**: LRT, likelihood-ratio test

**Supplementary Table S5. TNFi comparator - pooled vs etanercept only**

| **Outcome** | **Time point** | **TNFi total (mean +/- SD, n)** | **Etanercept only (mean +/- SD, n)** |
| --- | --- | --- | --- |
| **Current pain (NRS)** | Baseline | 5.9 +/- 2.3 (28) | 6.3 +/- 1.9 (23) |
|  | 2 weeks | 4.5 +/- 2.3 (28) | 4.8 +/- 2.0 (24) |
|  | 3 months | 4.2 +/- 2.8 (26) | 4.1 +/- 2.7 (23) |
| **Average pain (NRS)** | Baseline | 6.4 +/- 1.7 (28) | 6.4 +/- 1.7 (23) |
|  | 2 weeks | 4.9 +/- 2.5 (28) | 5.2 +/- 2.2 (24) |
|  | 3 months | 4.6 +/- 2.9 (26) | 4.6 +/- 2.8 (23) |
| **Worst pain (NRS)** | Baseline | 8.0 +/- 1.7 (28) | 7.8 +/- 1.8 (23) |
|  | 2 weeks | 6.1 +/- 2.8 (28) | 6.5 +/- 2.3 (24) |
|  | 3 months | 5.3 +/- 3.2 (26) | 5.4 +/- 3.2 (23) |
| **DAS28-ESR** | Baseline | 5.6 +/- 1.1 (31) | 5.5 +/- 1.0 (26) |
|  | 2 weeks | 4.2 +/- 1.3 (25) | 4.2 +/- 1.1 (21) |
|  | 3 months | 4.1 +/- 1.6 (26) | 4.0 +/- 1.7 (23) |

Descriptive trajectories (mean +/- SD) for the full TNFi group and for etanercept-treated patients only. The TNFi group comprised 26 etanercept and 5 adalimumab-type patients (3 adalimumab, 2 biosimilar).

**Supplementary Table S6. Baseline-adjusted cytokine trajectories**

| **Cytokine** | **Patients** | **Baseline-adjusted interaction p** |  |
| --- | --- | --- | --- |
| **log IL-6** | 52 | 0.128 |  |
| **log IL-12p40** | 52 | 0.305 |  |
| **log IL-12p70** | 52 | 0.093 |  |

Longitudinal mixed-effects models for the follow-up visits (2 weeks, 3 months) with the log-transformed baseline concentration of the respective cytokine as a covariate. The group-by-time interaction tests whether trajectories differed between groups after baseline adjustment.

**SUPPLEMENTARY FIGURES**

**S1: Spaghetti-Plots biomarker concentrations, baricitinib group**

**(A)**

**(B)**

**(C)**

**Supplementary Figure S1.** Individual patient trajectories for serum IL-6 (A), IL-12p40 (B) and IL-12p70 (C) in the baricitinib group at baseline, 2 weeks and 3 months. Each thin line represents one patient; the bold line indicates the group median. Concentrations are shown on a logarithmic scale owing to the right-skewed distributions. The plots illustrate the marked interindividual variability in cytokine responses. Missing values were not imputed; only available observations are connected.

**S2: Spaghetti-Plots biomarker concentrations, TNFi group**

**(A)**

**(B)**

**(C)**

**Supplementary Figure S2.** Individual patient trajectories for serum IL-6 (A), IL-12p40 (B) and IL-12p70 (C) in the TNFi group at baseline, 2 weeks and 3 months. Each thin line represents one patient; the bold line indicates the group median. Concentrations are shown on a logarithmic scale owing to the right-skewed distributions. The plots illustrate the marked interindividual variability in cytokine responses. Missing values were not imputed; only available observations are connected.
